# Supplementary material for: The neural basis of resting-state fMRI functional connectivity in fronto-limbic circuits revealed by chemogenetic manipulation
Source: Nat Commun. 2024 May 31;15:4669. doi: 10.1038/s41467-024-49140-0 (PMC11143237; doi:10.1038/s41467-024-49140-0)
Supplement: Supplementary file 1 — Supplementary Information [file 41467_2024_49140_MOESM1_ESM.pdf]

Supplementary Information for

The neural basis of resting-state fMRI functional connectivity in fronto-limbic circuits revealed by chemogenetic manipulation

Author list: Catherine Elorette[1,2#], Atsushi Fujimoto[1,2#], Frederic M. Stoll[1,2], Satoka H. Fujimoto[1,2], Niranjana Bienkowska[1,2], Liza London[1,2], Lazar Fleysher[3], Brian E. Russ[1,4,5+*], and Peter H. Rudebeck[1,2+*]

# These authors contributed equally: Catherine Elorette, Atsushi Fujimoto

+ These authors jointly supervised this work: Brian E. Russ, Peter H. Rudebeck

*Correspondence should be addressed to: brian.russ@nki.rfmh.org or peter.rudebeck@mssm.edu

Affiliations:

[1] Nash Family Department of Neuroscience and Friedman Brain Institute, Icahn School of Medicine at Mount Sinai, One Gustave L. Levy Place, New York, NY 10029, USA

[2] Lipschultz Center for Cognitive Neuroscience, Icahn School of Medicine at Mount Sinai, One Gustave L. Levy Place, New York, NY 10029, USA

[3] BioMedical Engineering and Imaging Institute, Icahn School of Medicine at Mount Sinai, One Gustave L. Levy Place, New York, NY 10029, USA

[4] Center for Biomedical Imaging and Neuromodulation, Nathan Kline Institute, 140 Old Orangeburg Road, Orangeburg, NY 10962, USA

[5] Department of Psychiatry, New York University at Langone, 550 1st Avenue, New York, NY 10016, USA

This file includes:

Supplementary Figures 1-6

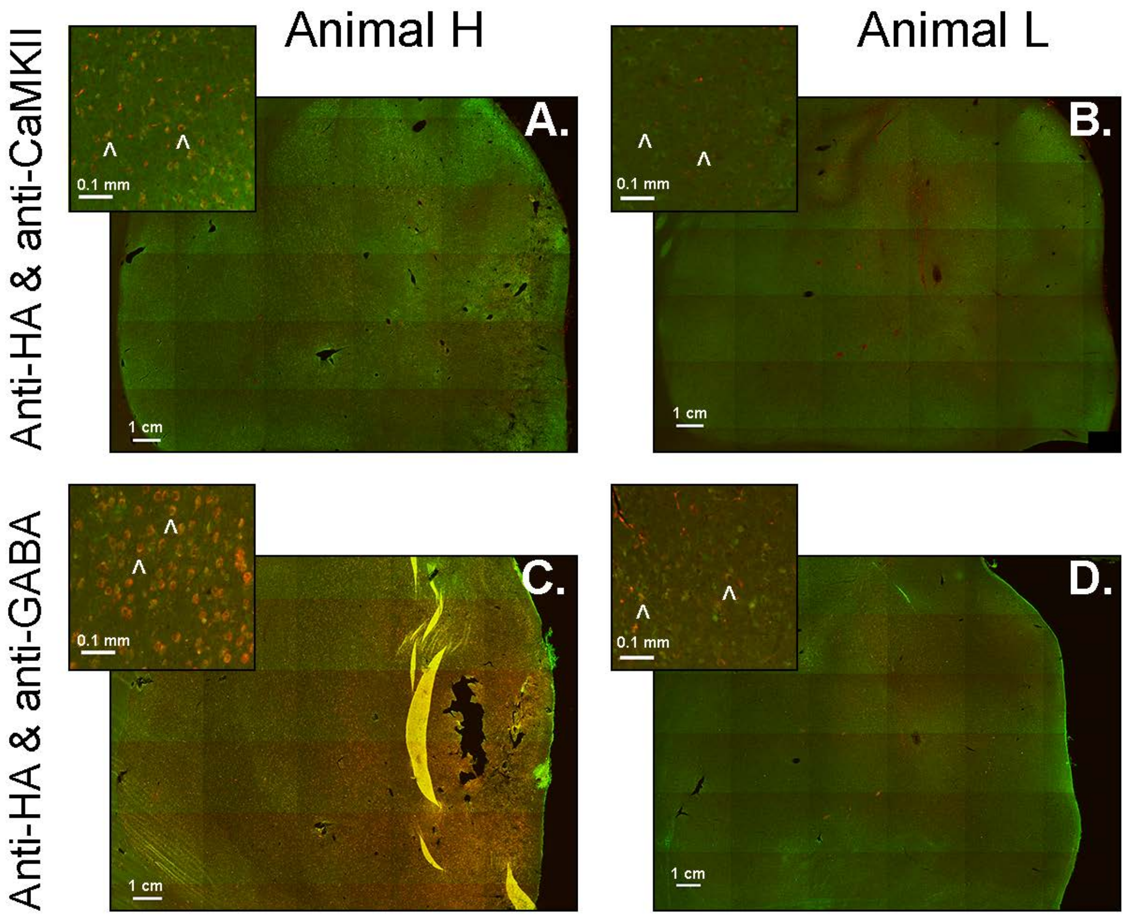

Supplementary Figure 1. Colocalization of DREADD receptors with markers for excitatory or inhibitory neurons in the amygdala. Left amygdala sections shown for animals H (left, A and C) and L (right, B and D) stained using an immunofluorescent approach. Images were taken at 5x magnification in a tiled arrangement and stitched together. Insets, top left, were taken at 10x magnification from the same section. Two white chevrons in each inset show examples of dual-labeled cells. **A-B.)** Sections stained for the DREADD receptor's HA tag (red) and CaMKII (green). Dual labeled cells appear yellow. At least 6 sections through the amygdala were stained for each animal. **C-D.)** Sections stained for the DREADD receptor's HA tag (red) and GABA (green). Dual labeled cells appear yellow. At least 6 sections through the amygdala were stained for each animal.

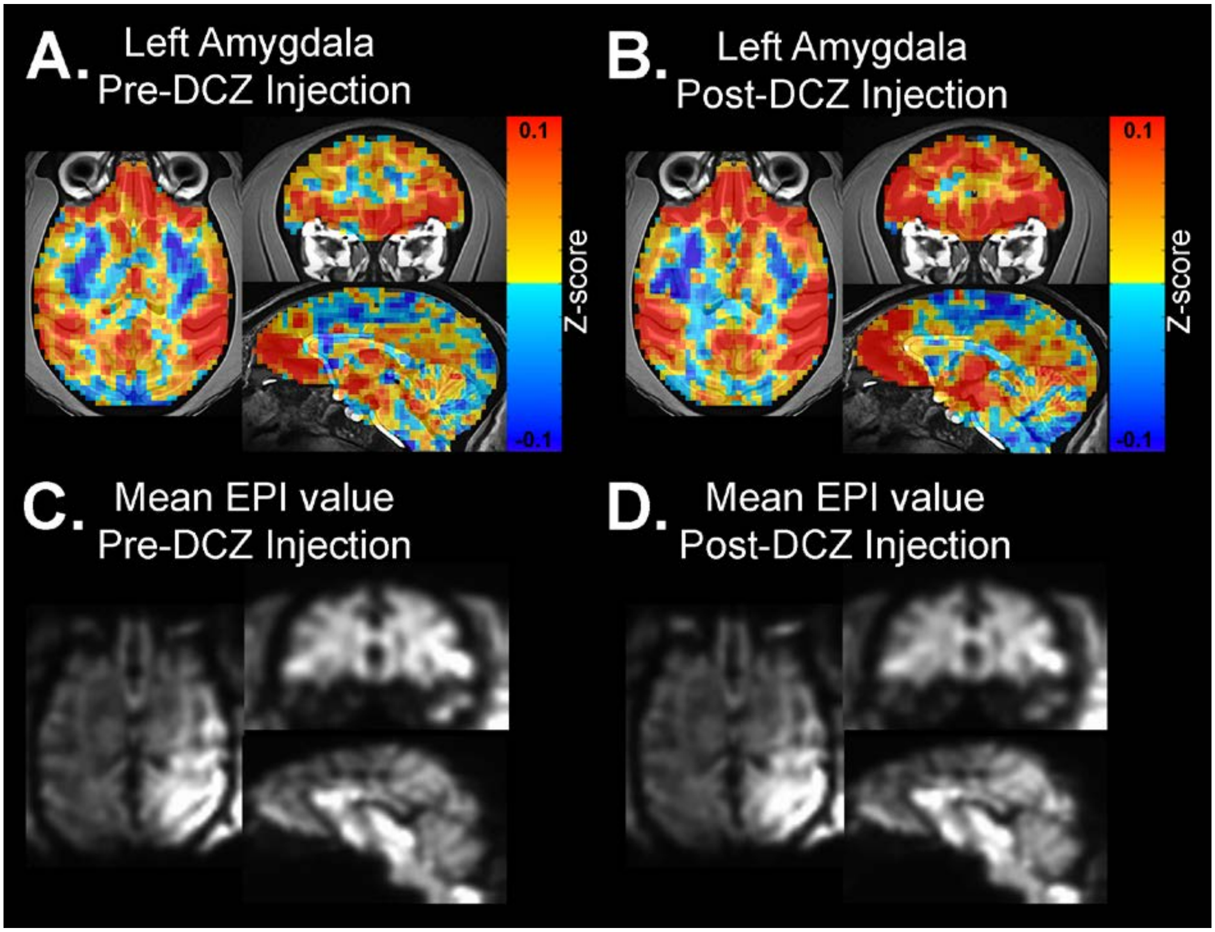

Supplementary Figure 2. Representative changes across a single imaging session before and after injection of DCZ. A-B) Changes in functional connectivity between amygdala and frontal cortex, unthresholded connectivity maps. Changes in FC with a left amygdala seed region in animal L in a single session, before and after DREADD activation with DCZ. Data shown on NMT[45]. Pre-DCZ injection period **(A)** and post-injection period **(B)**. Scale bar indicates z-score of rs-FC. No threshold or clustering has been applied. **C-D)** Average EPI values across the time series for the same session as in A-B. Pre-DCZ injection period, average across three EPI runs **(C)**, and post-injection period, average across three EPI runs **(D)**. Values were calculated after the blurring step of the preprocessing. These analyses were conducted for both hemispheres in both animals (N=2).

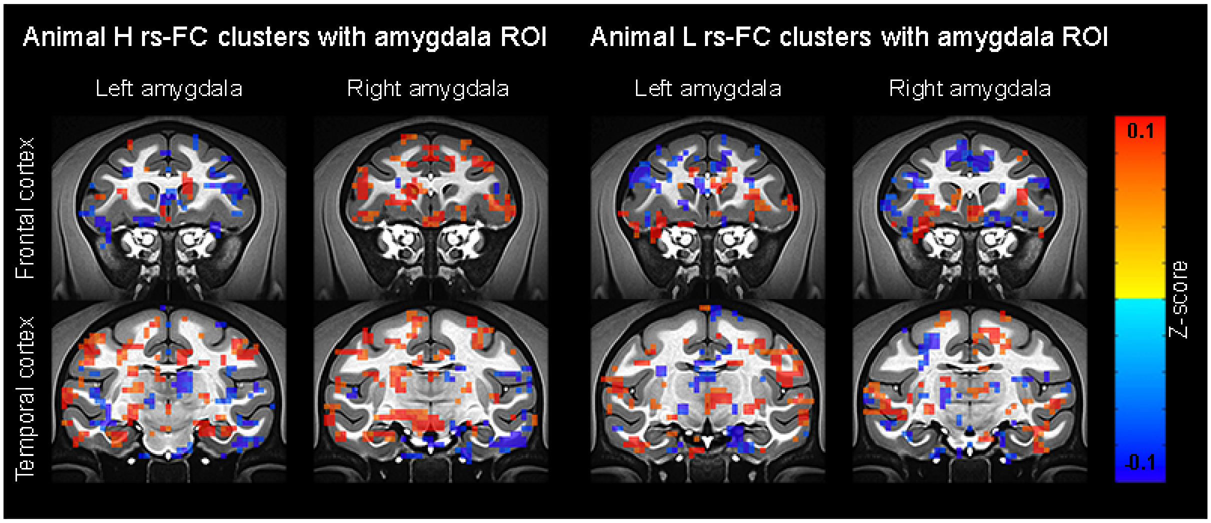

Supplementary Figure 3. Change in amygdala ROI rs-FC with frontal and temporal cortex after amygdala inhibition. Example coronal sections from each animal showing the change in rs-FC with a left or right amygdala seed after DREADD activation with DCZ, as compared to VEH. [(DCZ post-injection – pre-injection) – (VEH post-injection – pre-injection)], calculated from averaged DCZ post-injection – pre-injection data and averaged VEH post-injection – pre-injection data. Threshold p=0.05, cluster size ≥5 voxels, voxel faces touching. These analyses were conducted for both hemispheres in both animals (N=2). Data shown on NMT[45].

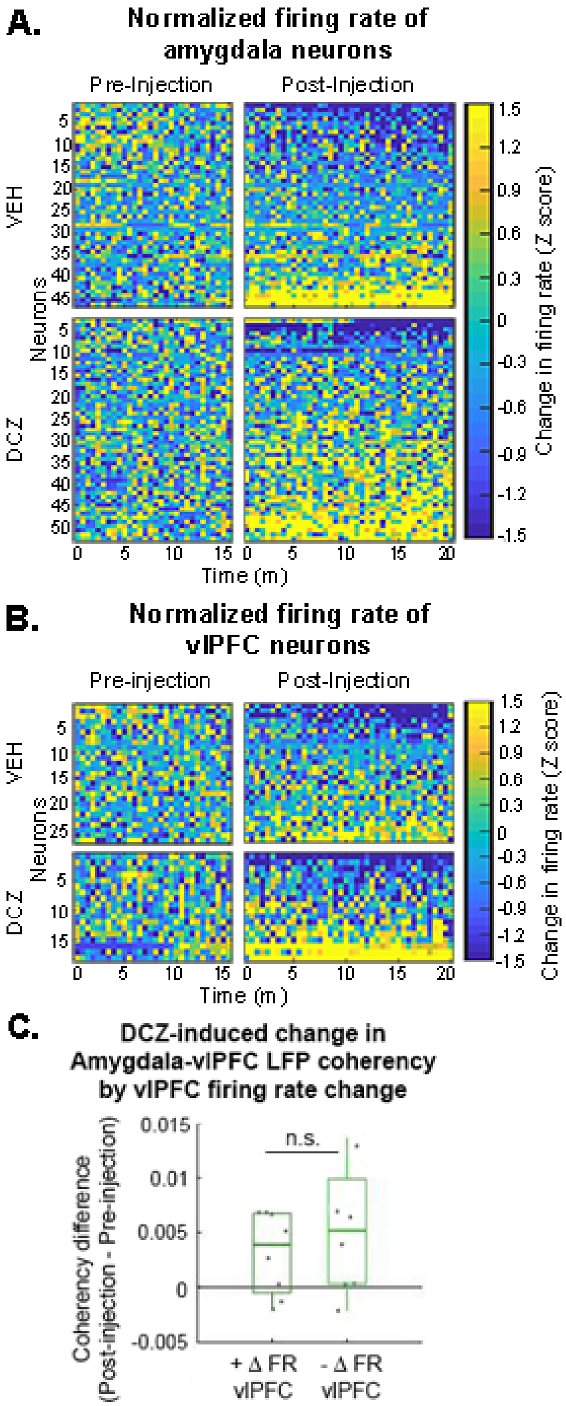

Supplementary Figure 4. Firing rate changes across time after activation of DREADDs with DCZ or after treatment with vehicle. Change in firing rate of each recorded neuron in the amygdala (**A**) and vlPFC (**B**), normalized to the average pre-injection firing rate, shown in thirty second bins. Data are separated by treatment (vehicle, top, or DCZ, bottom) and by recording period (pre-injection, left, 0-15 minutes, and post-injection, right, 0-20 minutes). Cool colors indicate a negative change in firing rate from the pre-injection period, warm colors indicate a positive change. **C**) Average changes in amygdala-vlPFC LFP coherency after DREADD inhibition with DCZ, grouped by changes in local firing rate in vlPFC. Grey dots indicate average coherency values between vlPFC bipolar sites where neurons were recorded and all amygdala bipolar sites. vlPFC bipolar sites where we recorded neurons that increased their firing rate after DCZ injection are shown on the left, dark green box plot; vlPFC bipolar sites where we recorded neurons that decreased their firing rate after DCZ injection are shown on the right, light green box plot. Kruskal-Wallis test, N=16, Chi2=0.47, p=0.49. Box plots display average value (center), spread of data from 25th to 75th percentiles (top and bottom of box), and maximal and minimal values (whiskers).

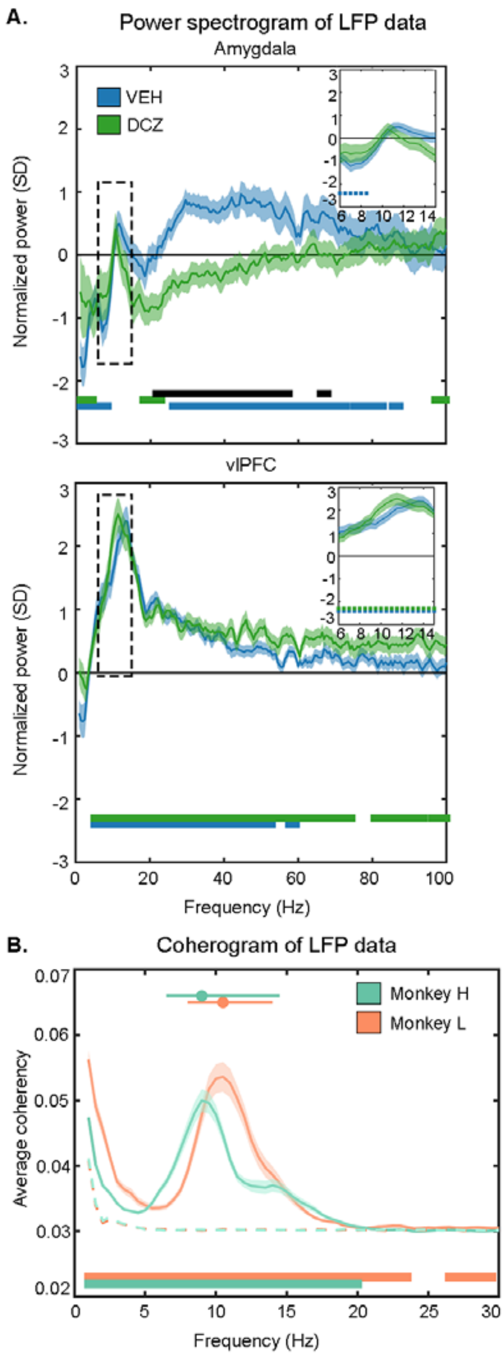

Supplementary Figure 5. Across the alpha band, coherency between amygdala and vlPFC is increased, but power is unaltered following DCZ treatment compared to vehicle. A) Post-injection power normalized to the pre-injection period, for the amygdala (top) and vlPFC (bottom) after treatment with DCZ or vehicle. Inset (top right corner, magnified from box) shows the power across the frequency band of interest used in the following analyses. Power is averaged across both animals. Dotted line (bottom of graph; blue for vehicle treatment, green for DCZ treatment) shows significant differences from pre-injection power (Wilcoxon signed rank test, $p<0.01$ for 5 consecutive frequency bins). Black dotted line (bottom of graph) indicates significant difference between DCZ and vehicle treatments (Kruskal-Wallis test, $p<0.01$ for 5 consecutive frequency bins). We observed a decrease in power in the beta-gamma range (20-60 Hz) after DCZ compared to vehicle treatment. No significant differences between DCZ and vehicle were observed in either region across the alpha band, the frequency of interest investigated in following analyses. **B**) Average coherency between amygdala and vlPFC bipolar sites across all periods and drug conditions for both animals (N=106). We observed a significant peak in coherency across the alpha band. Bottom lines indicate difference in coherency from randomized permutations (dotted line). Lines/circles (top) show the peak frequency + 1/3rd of the max value.

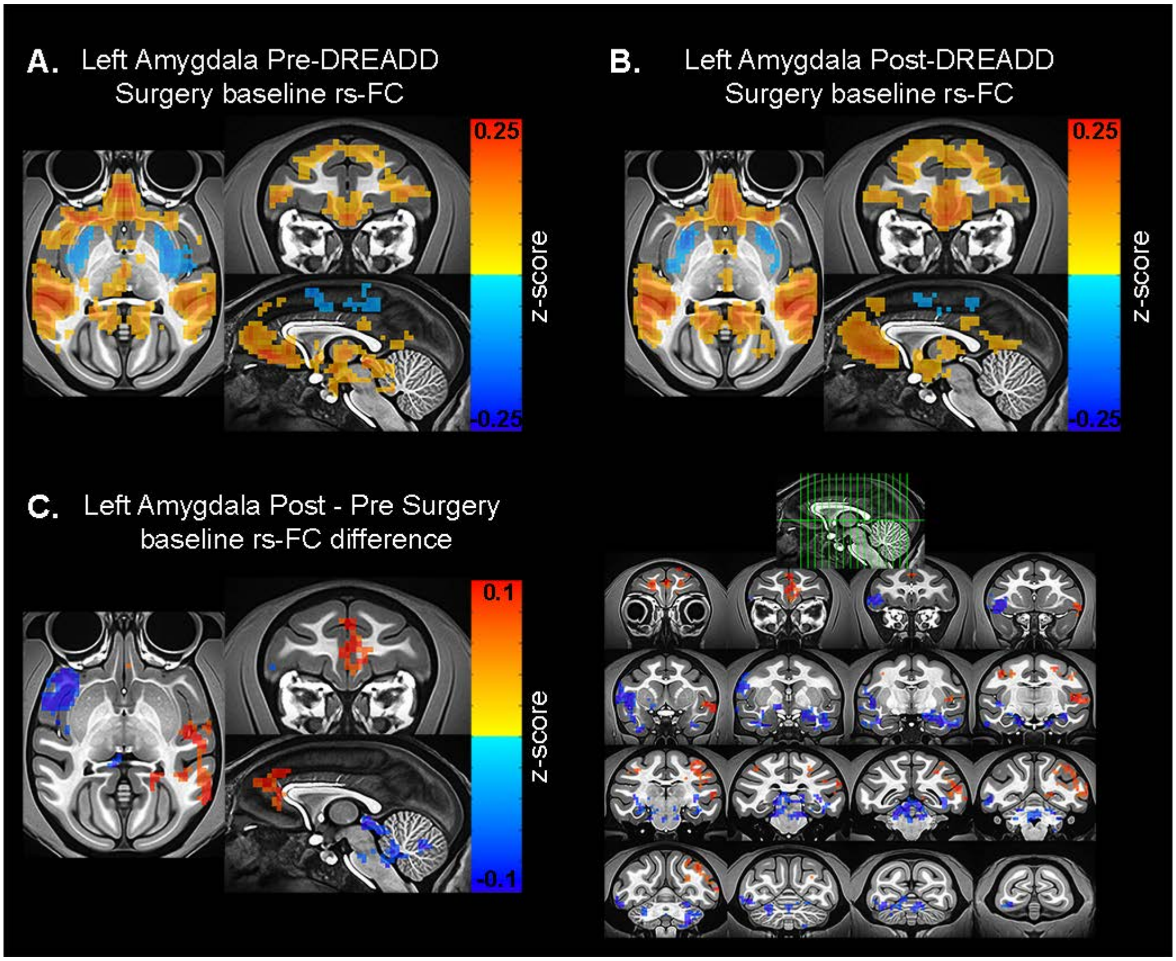

Supplementary Figure 6. Representative average amygdala rs-FC across the brain before and after DREADD transfection surgery. Average rs-FC with a left amygdala seed region in animal L. All data was taken from pre-drug injection period. **A.)** Pre-DREADD transfection surgery baseline, average of two sessions. Threshold p=0.0485, cluster size ≥30 voxels, voxel faces touching. r-value range -0.199:0.787. **B.)** Post-DREADD transfection surgery baseline, average of four sessions. Scale bar indicates z-score of rs-FC. Threshold p=0.0485, cluster size ≥30 voxels, voxel faces touching. Range of cluster correlation values -0.183:0.774. **C.)** Difference in rs-FC between post-DREADD transfection surgery baseline (B) and Pre-DREADD transfection surgery baseline (A). Scale bar indicates difference in z-score of rs-FC. Threshold p=0.0485, cluster size ≥30 voxels, voxel faces touching. r-value range -0.256:0.213. These analyses were conducted for both hemispheres in both animals (N=2). Data shown on NMT[45].
