# Supplementary material for: The neural basis of resting-state fMRI functional connectivity in fronto-limbic circuits revealed by chemogenetic manipulation
Source: Nat Commun. 2024 May 31;15:4669. doi: 10.1038/s41467-024-49140-0 (PMC11143237; doi:10.1038/s41467-024-49140-0)
Supplement: Supplementary file 3 — Reporting Summary [file 41467_2024_49140_MOESM3_ESM.pdf]

nature portfolio

Corresponding author(s): Peter Rudebeck, Brian Russ

Last updated by author(s): Apr 18, 2024

Reporting Summary

Nature Portfolio wishes to improve the reproducibility of the work that we publish. This form provides structure for consistency and transparency in reporting. For further information on Nature Portfolio policies, see our Editorial Policies and the Editorial Policy Checklist.

Statistics

For all statistical analyses, confirm that the following items are present in the figure legend, table legend, main text, or Methods section.

n/a	Confirmed	
☐	☒	The exact sample size (*n*) for each experimental group/condition, given as a discrete number and unit of measurement
☐	☒	A statement on whether measurements were taken from distinct samples or whether the same sample was measured repeatedly
☐	☒	The statistical test(s) used AND whether they are one- or two-sided<br>*Only common tests should be described solely by name; describe more complex techniques in the Methods section.*
☐	☒	A description of all covariates tested
☐	☒	A description of any assumptions or corrections, such as tests of normality and adjustment for multiple comparisons
☐	☒	A full description of the statistical parameters including central tendency (e.g. means) or other basic estimates (e.g. regression coefficient) AND variation (e.g. standard deviation) or associated estimates of uncertainty (e.g. confidence intervals)
☐	☒	For null hypothesis testing, the test statistic (e.g. F, t, r) with confidence intervals, effect sizes, degrees of freedom and P value noted<br>*Give P values as exact values whenever suitable.*
☒	☐	For Bayesian analysis, information on the choice of priors and Markov chain Monte Carlo settings
☐	☒	For hierarchical and complex designs, identification of the appropriate level for tests and full reporting of outcomes
☐	☒	Estimates of effect sizes (e.g. Cohen's d, Pearson's r), indicating how they were calculated

Our web collection on statistics for biologists contains articles on many of the points above.

Software and code

Policy information about availability of computer code

Data collection	PlexControl (20)
Data analysis	MATLAB (2022b), AFNI (21.0.08), SPM12, Fieldtrip toolbox (20221223 release)

For manuscripts utilizing custom algorithms or software that are central to the research but not yet described in published literature, software must be made available to editors and reviewers. We strongly encourage code deposition in a community repository (e.g. GitHub). See the Nature Portfolio guidelines for submitting code & software for further information.

Data

Policy information about availability of data

All manuscripts must include a data availability statement. This statement should provide the following information, where applicable:

- Accession codes, unique identifiers, or web links for publicly available datasets
- A description of any restrictions on data availability
- For clinical datasets or third party data, please ensure that the statement adheres to our policy

Provide your data availability statement here.

Research involving human participants, their data, or biological material

Policy information about studies with human participants or human data. See also policy information about sex, gender (identity/presentation), and sexual orientation and race, ethnicity and racism.

Reporting on sex and gender	This study did not involve human subjects.
Reporting on race, ethnicity, or other socially relevant groupings	This study did not involve human subjects.
Population characteristics	This study did not involve human subjects.
Recruitment	This study did not involve human subjects.
Ethics oversight	This study did not involve human subjects.

Note that full information on the approval of the study protocol must also be provided in the manuscript.

Field-specific reporting

Please select the one below that is the best fit for your research. If you are not sure, read the appropriate sections before making your selection.

☒ Life sciences ☐ Behavioural & social sciences ☐ Ecological, evolutionary & environmental sciences

For a reference copy of the document with all sections, see nature.com/documents/nr-reporting-summary-flat.pdf

Life sciences study design

All studies must disclose on these points even when the disclosure is negative.

Sample size	The sample sizes were determined using standard practices in the field for working with macaques that balance the ethical and practical constraints of conducting research in non-human primates. We referred to previous studies with similar methodologies when determining sample size (Turchi et al. Neuron 2018, Hirabayashi et al. Neuron 2021, Froesel et al. Nature Communications 2022, Liu et al. Nature Communications 2022, Noritake et al. Nature Communications 2023).
Data exclusions	Neurophysiology data were excluded if there was excessive electrical noise on the recording electrodes. Otherwise no selection criteria were applied.
Replication	Our analyses were conducted within subject meaning that each animal served as their own control. We conducted two repeated sessions of fMRI and neurophysiology data acquisition for both DCZ and vehicle treatments in both animals in order to increase the power. Further, our units of analysis were at the level of neurons and voxels, which builds in replication to our design.
Randomization	Monkeys were randomly assigned to the study. Further all neurons recorded in prefrontal cortex and amygdala were randomly selected.
Blinding	Cell counts/stereology of DREADD positive neurons was conducted blind to the results of the fMRI and neurophysiology components. fMRI and neurophysiology data collection and analysis were not performed blind due to the limitations of staff cleared to work with non-human primates. Analyses performed in MATLAB and AFNI were conducted using standard scripts applied uniformly to all data, reducing the need for blinding.

Reporting for specific materials, systems and methods

We require information from authors about some types of materials, experimental systems and methods used in many studies. Here, indicate whether each material, system or method listed is relevant to your study. If you are not sure if a list item applies to your research, read the appropriate section before selecting a response.

Materials & experimental systems

n/a	Involved in the study
☐	☒ Antibodies
☒	☐ Eukaryotic cell lines
☒	☐ Palaeontology and archaeology
☐	☒ Animals and other organisms
☒	☐ Clinical data
☒	☐ Dual use research of concern
☒	☐ Plants

Methods

n/a	Involved in the study
☒	☐ ChIP-seq
☒	☐ Flow cytometry
☐	☒ MRI-based neuroimaging

Antibodies

Antibodies used

HA-Tag (C29F4) Rabbit mAb antibody, Cell Signaling Technology, cat # 3724, lot # 11, RRID:AB_1549585
Goat Anti-Rabbit IgG Antibody (H+L), Biotinylated, Vector Laboratories, cat # BA-1000, lot # ZH1221, RRID:AB_2313606
VECTASTAIN Elite ABC-Peroxidase Kit, Vector Laboratories, cat # PK-6100, lot # ZJ0111, RRID:AB_233681
Anti-CaM Kinase II Antibody, α subunit, clone 6G9, Sigma-Aldrich, cat # 05-532, lot # 3861317, RRID:AB_309787
Monoclonal Anti-GABA Antibody, clone GB-69, Sigma-Aldrich, cat # A0310, source # 0000143053, RRID:AB_476667
Donkey anti-Rabbit IgG (H+L) Highly Cross-Adsorbed Secondary Antibody, Alexa Fluor™ 647, Invitrogen, cat # A31573, lot # 2420695, RRID:AB_2536183
Goat anti-Mouse IgG (H+L) Highly Cross-Adsorbed Secondary Antibody, Alexa Fluor™ Plus 488, Invitrogen, cat # A32723, lot # XG349343, RRID:AB_2633275

Validation

All antibodies were validated by the manufacturers and validation data are publicly available. See manufacturer's website or Research Resource Identification Portal for citations to published works using these resources.

Animals and other research organisms

Policy information about studies involving animals; ARRIVE guidelines recommended for reporting animal research, and Sex and Gender in Research

Laboratory animals

2 Macaca mulatta, male, age 7

Wild animals

This study did not involve wild animals.

Reporting on sex

To the best of our knowledge, findings in this study reflect general neural mechanisms that should apply to both male and female animals. Sex was therefore not considered as a variable in the experimental design.

Field-collected samples

This study did not involve samples collected from the field.

Ethics oversight

Icahn School of Medicine Animal Care and Use Committee

Note that full information on the approval of the study protocol must also be provided in the manuscript.

Plants

Seed stocks

Report on the source of all seed stocks or other plant material used. If applicable, state the seed stock centre and catalogue number. If plant specimens were collected from the field, describe the collection location, date and sampling procedures.

Novel plant genotypes

Describe the methods by which all novel plant genotypes were produced. This includes those generated by transgenic approaches, gene editing, chemical/radiation-based mutagenesis and hybridization. For transgenic lines, describe the transformation method, the number of independent lines analyzed and the generation upon which experiments were performed. For gene-edited lines, describe the editor used, the endogenous sequence targeted for editing, the targeting guide RNA sequence (if applicable) and how the editor was applied.

Authentication

Describe any authentication procedures for each seed stock used or novel genotype generated. Describe any experiments used to assess the effect of a mutation and, where applicable, how potential secondary effects (e.g. second site T-DNA insertions, mosiacism, off-target gene editing) were examined.

Magnetic resonance imaging

Experimental design

Design type

Resting-state

Design specifications

Each resting state imaging session consisted of three approximately 10-minute/300 volume runs as control data,

Design specifications	followed by administration of drug and another three approximately 10-minute/300 volume runs of experimental data.
Behavioral performance measures	This study did not involve behavioral tasks.

Acquisition

Imaging type(s)	Functional, structural
Field strength	3T
Sequence & imaging parameters	Structural: T1-weighted, 0.5 mm isotropic, FOV 288 x 288, Matrix size 288 x 288, TR/TE 2500/2.81 ms, flip angle 8°, coronal orientation<br>Functional: Echo planar image, 1.6 mm isotropic, FOV 602 x 602, Matrix size 86 x 86, TR/TE 2120/16ms, flip angle 45°, coronal orientation
Area of acquisition	Whole brain

Diffusion MRI ☐ Used ☒ Not used

Preprocessing

Preprocessing software	AFNI
Normalization	The T1-weighted images were spatially normalized, then skull-stripped using the U-Net model built from Primate Data-Exchange open datasets
Normalization template	NMT version 2.0 atlas
Noise and artifact removal	Blur: FWHM of 3 mm
Volume censoring	The first two TRs of each functional scan were removed to avoid magnetization effects on the data. The regress_censor_motion (limit 0.10) and regress_censor_outliers (limit 0.02) settings in AFNI's afni_proc.py function were used to censor volumes with excess motion.

Statistical modeling & inference

Model type and settings	Where appropriate we used parametric, non-parametric and count based statistics to analyze our data.
Effect(s) tested	We tested for effects between the administration of vehicle versus DCZ or vehicle versus CNO across our fMRI and neurophysiology data (both single neuron and local field potentials).

Specify type of analysis: ☐ Whole brain ☒ ROI-based ☐ Both

Anatomical location(s)	All ROIs were taken from standard macaque templates (for example, D99).
Statistic type for inference (See Eklund et al. 2016)	Voxel-wise false discovery rate
Correction	Cluster correction using AFNI's 3DClusterize function

Models & analysis

n/a	Involved in the study
☐	☒ Functional and/or effective connectivity
☒	☐ Graph analysis
☒	☐ Multivariate modeling or predictive analysis

Functional and/or effective connectivity	Pearson correlation
